# Effects of neuromuscular electrostimulation in patients with heart failure admitted to ward

**DOI:** 10.1186/1749-8090-7-124

**Published:** 2012-11-15

**Authors:** Carlos José Soares de Araújo, Fernanda Souza Gonçalves, Hugo Souza Bittencourt, Noélia Gonçalves dos Santos, Sérgio Vitor Mecca Junior, Júlio Leal Bandeira Neves, André Maurício Souza Fernandes, Roque Aras Junior, FranciscoJoséFariasBorges dos Reis, Armênio Costa Guimarães, ErenaldodeSouzaRodrigues Junior, Vitor Oliveira Carvalho

**Affiliations:** 1 Hospital Ana Neri, Universidade Federal da Bahia, Avenida Saldanha Marinho s/n, Salvador Bahia, CEP:40320-010, Brazil; 2 Faculdade de Medicina da Universidade Federal da Bahia (UFBa), Salvador-BA, Brazil; 3 Unidade de Cirurgia Cardíaca Pediátrica do Instituto do Coração do Hospital das Clínicas da Faculdade de Medicina da USP, São Paulo, Brazil; 4 The GREAT Group (GRupo de Estudos em Atividade física), São Paulo, Brazil

**Keywords:** Heart failure, Exercise, Physiotherapy, Rehabilitation, Neuromuscular electrostimulation

## Abstract

**Background:**

Neuromuscular electrostimulation has become a promising issue in cardiovascular rehabilitation. However there are few articles published in the literature regarding neuromuscular electrostimulation in patients with heart failure during hospital stay.

**Methods:**

This is a randomized controlled pilot trial that aimed to investigate the effect of neuromuscular electrostimulation in the walked distance by the six-minute walking test in 30 patients admitted to ward for heart failure treatment in a tertiary cardiology hospital. Patients in the intervention group performed a conventional rehabilitation and neuromuscular electrostimulation. Patients underwent 60 minutes of electrostimulation (wave frequency was 20 Hz, pulse duration of 20 us) two times a day for consecutive days until hospital discharge.

**Results:**

The walked distance in the six-minute walking test improved 75% in the electrostimulation group (from 379.7 ± 43.5 to 372.9 ± 46.9 meters to controls and from 372.9 ± 62.4 to 500 ± 68 meters to electrostimulation, p<0.001). On the other hand, the walked distance in the control group did not change.

**Conclusion:**

The neuromuscular electrostimulation group showed greater improvement in the walked distance in the six-minute walking test in patients admitted to ward for compensation of heart failure.

## Background

Heart failure is the last stage of heart diseases and a significant cause of worldwide mortality and morbidity [1]. It is characterized by exercise intolerance, [2] high mortality [3] and poor quality of life [4].

Exercise training is considered to be an important and safe component of a heart failure rehabilitation program. This therapy is endorsed by current guidelines, [5,6] and patients who join an exercise training program are expected to improve exercise capacity [7] and quality of life [8].

Neuromuscular electrostimulation is largely used as an alternative adjuvant tool of exercise training to treat muscle atrophy secondary to disuse in healthy people and in patients with neuromuscular disorders [9]. This technique has become a promising issue in cardiovascular rehabilitation and a growing number of studies have being released with good results.

Initial studies showed that neuromuscular electrostimulation can improve peak oxygen consumption [10] and fatigue tolerance in patients with heart failure [11]. It seems that the best indication of neuromuscular electrostimulation is for those patients that cannot perform the conventional rehabilitation [12]. However there are no articles published in the literature regarding neuromuscular electrostimulation in patients with heart failure during hospital stay.

The aim of this randomized controlled pilot trial was to investigate the influence of neuromuscular electrostimulation in the walked distance in the six-minute walking test of heart failure patients during hospital stay.

## Methods

### Trial design

This is a randomized controlled pilot trial that aimed to investigate the effects neuromuscular electrostimulation in patients admitted to ward for treatment of decompensated heart failure.

As soon as the physician authorized the patients to be included in the protocol, one independent blinded investigator performed a six-minute walking test followed by blood sample collection (blood venous gas analysis and lactate). After initial assessment, patients were randomized to neuromuscular electrostimulation or control groups by a second investigator. Immediately before hospital discharge, patients repeated the same assessment protocol by the same blinded investigator.

### Study population

**Figure 1:**
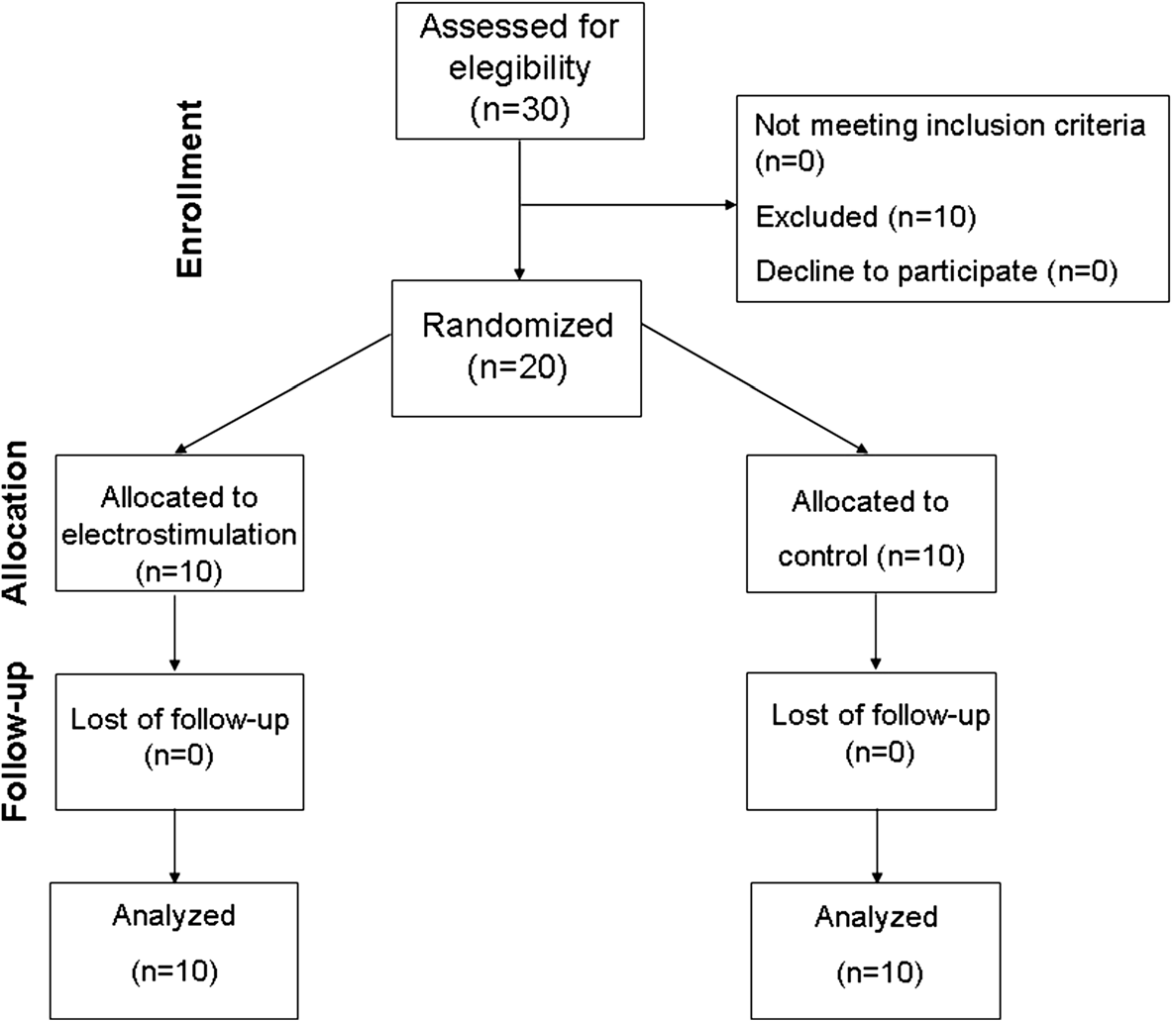
Participants flow through the study.

We included patients with heart failure aged over 18 years, with left ventricular ejection fraction less than 45% (echocardiography) admitted to ward in a tertiary cardiology hospital for treatment of decompensated heart failure. The characteristics of the subjects and their medication profiles at the inclusion are shown in Table 1. Patients with New York Heart Association Functional Class IV, previous experience with neuromuscular electrostimulation, with fever, using vasoactive drugs, in program for surgery and patients with osteopathic and pulmonary function limitations such as osteoarthritis and chronic obstructive pulmonary disease were excluded from the study (Figure 1).

**Table 1:** Patient’s characterization and medication profile

	**Control**	**Intervention**
Etiology N (%)		
Ischemic	04 (40%)	04 (40%)
Non Ischemic	06 (60%)	06 (60%)
NYHA functional class (%)		
I	0	0
II	06 (60%)	06 (60%)
III	04 (40%)	04 (40%)
Sex (%)		
Male	06 (60%)	06 (60%)
Female	04 (40%)	04 (40%)
Age (years, mean ±SD)	49,5±14,3	52,2±9
LVEF (%, mean ±SD)	38,2±8,8	37,6±6,9
Current medications:		
Diuretics	10 (100%)	10 (100%)
Digitalis	05 (50%)	04 (40%)
Betablocker	10 (100%)	09 (90%)
Vasodilator	02 (20%)	04 (40%)
Ca^2+^channel blocker	01 (10%)	01 (10%)
Anti-arrhythmic	01 (10%)	---

NYHA= New York Heart Association, LVEF= Left ventricular ejection fraction (echo), ACE= Angiotensin Converting Enzyme.

This protocol was approved by the Ethical Committee of our institution. All patients provided informed consent prior to participation.

### End points

The primary endpoint of this study was the walked distance in the six-minute walking test. The secondary endpoints were venous blood lactate and venous saturation of oxygen before and after neuromuscular electrostimulation.

### Randomization

After providing written informed consent, patients were randomized in a 1:1 ratio to electrostimulation or control. To guarantee concealment of the allocation list, randomization was implemented through a Web-based automated randomization system.

### Blinding

Considering our intervention protocol, it was not possible to blind the patients and/or the investigator who performed the electrostimulation. However, the other investigator who conducted the six-minute walking tests was blinded.

### Study interventions

Patients in the intervention group underwent a conventional rehabilitation and neuromuscular electrostimulation. Conventional rehabilitation consisted in respiratory, lower and upper limbs exercises in a stand position (3x10 repetitions, “‘somewhat hard–hard’, between 11 and 13 on the Borg scale).

Neuromuscular electrostimulation was applied by an electrostimulator (Neurodim III, IBRAMED, Brazil) with alternating, biphasic and symmetric currents with rectangular pulses. The carrier wave frequency was 20 Hz, pulse duration of 200 us. The time of ascent and descent of the current was 4 s and the time of contraction/relaxation was 20 s. Adhesive surface electrodes 5 cm in diameter were used through 4 channels in *rectus femoris* (2 channels in the right and 2 in the left leg).

Patients underwent 60 minutes of electrostimulation two times a day (morning and afternoon) for consecutive days until hospital discharge. Patients were instructed not to perform rehabilitation exercises during the neuromuscular electrostimulation sessions.

### Control group

The control group performed the same conventional rehabilitation protocol as the intervention group, but the electrostimulator device was turned off.

### Six-minute walking test

The six-minute walking test was performed following the American Thoracic Society guidelines [13] in a 20-meters corridor. The first test was performed after the medical team’s authorization and the second test was performed immediately before patient’s hospital discharge.

### Blood lactate and venous oxygen saturation

A blood sample of 01 ml was collected in patient’s peripheral venous access from the catheter line immediately after the six-minute walking test. Blood lactate and venous oxygen saturation were analyzed through Roche OMNI® S Blood Gas Analyzer (Roche Diagnostics, USA).

### Statistical analysis

The results are shown as mean and standard deviation. To evaluate the effects of the electrostimulation between both groups, we used analysis of variance (ANOVA). Data was analyzed using the Statistical Package for Social Sciences for Windows, 11.5 (SPSS Inc, Chicago, Illinois, USA). Statistical significance was defined as p < 0.05.

## Results

### Study participants

Between July 2011 and October 2011, 30 patients were enrolled in a tertiary cardiology hospital in Brazil. Ten patients did not meet inclusion criteria (Two patients needed vasoative drugs / intensive care unit, three had fever, three arrhythmia and two patients underwent cardiac surgery). Ten patients were allocated to electrostimulation and ten to placebo groups and they all were analyzed for the primary outcome (Figure 1). Our sample did not show losses or exclusions after randomization. The baseline characteristics were well balanced between the groups (Table 1). The studied patients did not show any harms or unintended effects, such as muscular pain. The average of exercise sessions in the electrostimulation and control groups was 16±3 and 15±4 days respectively.

**Figure 2:**
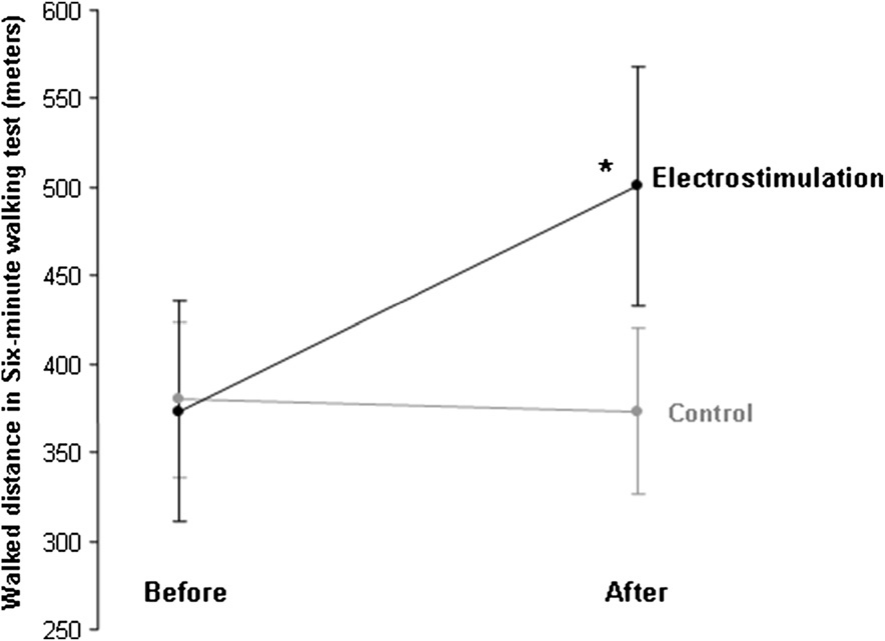
Walked distance in six-minute walking test (meters) before and after neuromuscular electrostimulation (*p<0.001). Data are presented as mean ± standard deviation.

Our primary end-point, the walked distance in the six-minute walking test, showed a 75% of improvement in the electrostimulation group (p<0.001) (Figure 2). On the other hand, the walked distance in the control group did not change (Table 2).

**Table 2:** Effects of neuromuscular electrostimulation in heart failure patients

	**Control (mean±SD)**		**Intervention (mean±SD)**		***p***
	**Before**	**After**	**Before**	**After**	
**Distance in 6MWT (meters)**	379.7 ± 43.5	372.9 ± 46.9	372.9 ± 62.4	500 ± 68	<0.001
**SvO**_**2**_**(%)**	84.3 ± 3.3	85.9 ± 3.0	88 ± 3.6	78,4 ± 3,8	<0.001
**Lactate (mmol/L)**	2.59 ± 0.7	2.5 ± 0.7	3.0 ± 0.9	2.0 ± 0,2	<0.001

*6MWT= six-minute walking test,* SvO_2_ = venous oxygen saturation.

Our secondary end-points showed the same behavior than the walked distance in the six-minute walking test. Patients in the electrostimulation group decreased the blood lactate in 34% and venous saturation of oxygen in 12% (p<0.001) (Table 2). The control group did not show differences in the blood lactate and in venous saturation of oxygen.

## Discussion

The main finding of this randomized controlled pilot trial was the improvement in the walked distance in the six-minute walking test after neuromuscular electrostimulation. Moreover, patients showed decrease in venous oxygen saturation and in blood lactate.

The relationship between heart failure, muscle impairment and low exercise capacity is well known in cardiology. Neuromuscular electrostimulation is largely used to treat muscle atrophy secondary to disuse in healthy people and in patients with neuromuscular disorders.^9^ Thus, neuromuscular electrostimulation has been proposed as a promising adjuvant therapy to potentialize the effects of exercise training in patients engaged in cardiovascular rehabilitation programs. Nevertheless, this therapy seems to be well indicated in patients with major functional limitations who can not perform the conventional physical exercise training program (such as bedridden patients). [14] On the other hand, our pilot study showed a large benefit of using neuromuscular electrostimulation in patients admitted to ward that were able to perform the conventional phase I rehabilitation program.

Two previous studies used a similar electrostimulation protocol as ours for outpatients with heart failure and showed good results in the walked distance in the six-minute walking test., [15,16] In the study Nuhr et al.,^16^ the walked distance was much greater, which suggests that the duration of the electrostimulation protocol can play an important role in cardiovascular rehabilitation.

A meta-analysis showed that patients with greater functional limitation are the most benefited with neuromuscular electrostimulation. On the other hand, our study showed that patients with lower exercise intolerance also seem to benefit from electrostimulation. The number of studies that compare the effects of neuromuscular electrostimulation in heart failure patients at different functional status and perform the same protocol is very limited, making it difficult to interpret the data.

Although our results are promising, we can not indicate neuromuscular electrostimulation to all patients with heart failure admitted to ward for treatment of decompensated heart failure. Our study suggests a new discussion about the indications of neuromuscular electrostimulation in patients with heart failure, especially for those patients admitted to ward. This adjunct therapy is relatively costly and a wide investigation is needed to elucidate which patients are most suitable to this treatment.

The main limitation of this pilot trial was the sample size. However, our data is very consistence regarding the improvement of the walked distance in the six-minute walking test with the use of neuromuscular electrostimulation.

## Conclusion

The neuromuscular electrostimulation group showed a greater improvement in the walked distance in the six-minute walking test in comparison to the control group. Moreover, the intervention group showed a greater decrease in venous oxygen saturation and in blood lactate. Neuromuscular electrostimulation seems to be an important adjuvant therapy in a rehabilitation program for those patients admitted in ward for treatment of decompensated heart failure.

## Competing interests

The authors have no conflict of interest to disclose.

## Author’s contributions

CJSA, FSG, HSB, NGS, SVMJ, JLBN, AMSF, RAJ, FJFBR, ACG, VOC and ESRJ conceived the study and drafted the manuscript. CJSA, FSG and HSB performed the experiments. ESRJ and VOC analyzed the data. ESRJ supervised the experimental work. All authors have read and approved the final manuscript.
